# Prediction of Dangerous Driving Behavior Based on Vehicle Motion State and Passenger Feeling Using Cloud Model and Elman Neural Network

**DOI:** 10.3389/fnbot.2021.641007

**Published:** 2021-04-29

**Authors:** Huaikun Xiang, Jiafeng Zhu, Guoyuan Liang, Yingjun Shen

**Affiliations:** ^1^School of Automotive and Transportation Engineering, Shenzhen Polytechnic, Shenzhen, China; ^2^Center for Intelligent Biomimetic Systems, Shenzhen Institute of Advanced Technology, Chinese Academy of Sciences, Shenzhen, China; ^3^Guangdong Provincial Key Lab of Robotics and Intelligent System, Shenzhen Institutes of Advanced Technology, Chinese Academy of Sciences, Shenzhen, China; ^4^Shenzhen Key Laboratory of Human-Machine Intelligence-Synergy Systems, Shenzhen Institutes of Advanced Technology, Chinese Academy of Sciences, Shenzhen, China; ^5^Guangdong-Hong Kong-Macao Joint Laboratory of Human-Machine Intelligence-Synergy Systems, Shenzhen Institutes of Advanced Technology, Chinese Academy of Sciences, Shenzhen, China; ^6^School of Business, Nanjing University, Nanjing, China

**Keywords:** dangerous driving behavior, cloud model, Elman neural network, auto driving scenarios, active vehicle safety management

## Abstract

Dangerous driving behavior is the leading factor of road traffic accidents; therefore, how to predict dangerous driving behavior quickly, accurately, and robustly has been an active research topic of traffic safety management in the past decades. Previous works are focused on learning the driving characteristic of drivers or depended on different sensors to estimate vehicle state. In this paper, we propose a new method for dangerous driving behavior prediction by using a hybrid model consisting of cloud model and Elman neural network (CM-ENN) based on vehicle motion state estimation and passenger’s subjective feeling scores, which is more intuitive in perceiving potential dangerous driving behaviors. To verify the effectiveness of the proposed method, we have developed a data acquisition system of driving motion states and apply it to real traffic scenarios in ShenZhen city of China. Experimental results demonstrate that the new method is more accurate and robust than classical methods based on common neural network.

## Introduction

Driving behavior analysis is an important part of research on traffic safety, which is a reflection of how the driver steers the vehicle including speed and attitude control. Dangerous driving behaviors are seen as series of operations performed by the driver on public roads that may result in abnormal traffic conditions and subsequently lead to road accidents (Dronseyko et al., 2018). Therefore, the analysis of driving behavior can help to measure the driver’s driving safety and prevent traffic accidents. A recent report by the American Automobile Association estimated that 56% of fatal crashes occurring between 2003 and 2007 are related to aggressive driving behavior (American Automobile Association, 2009). In Shanghai, China, traffic police corps reported that 75.9% (792 out of 1,044) of the car accidents in 2015 were caused by all kinds of dangerous driving behaviors (Accident Prevention Division of Traffic Police Corps of Shanghai Public Security Bureau, 2016). If the dangerous driving behavior of vehicles can be identified in time, the driver may be promptly alerted or the vehicle may be forcibly taken over at a critical time by safety control devices, which will effectively prevent the traffic accidents from happening.

Case by case modeling driver’s personal driving behavior is the most straightforward way; however, dangerous driving behavior involves various complex and uncertain factors, such as driving skills, emergency response ability, gender, mood, fatigue, job pressure and even educational background, life experience, etc. (Horswill and McKenna, 1999; Harre and Sibley, 2007; Dula et al., 2011; Day et al., 2018; Fountas et al., 2019; Useche et al., 2020), thereby making it difficult to directly study personal driving behavior. Nevertheless, during the course of driving, no matter how complex factors the vehicle is subjected to and no matter what driving actions the driver takes, all dangerous driving behaviors will eventually be reflected through the corresponding motion state of vehicle and reaction of passengers on the vehicle. Therefore, we can detect potential dangerous driving behavior by sensing vehicle motion explicitly and monitoring passenger’s feeling implicitly. Based on this fact, this paper intends to use real-time monitoring data, including explicit vehicle states and implicit passenger feelings to study dangerous driving behavior.

The main contributions of this paper are as follows: (1) Passenger feeling scores are introduced into the prediction system as subjective evaluations on the driver’s behaviors; (2) cloud model (CM) is applied to identify the state of vehicle with a clear qualitative judgment, and combined with Elman neural network to make predictions; (3) a complete and practical solution including hardware and algorithms is presented for the prediction of dangerous driving behaviors.

## Related Work

In the research of driving behavior analysis based on real-time monitoring data of vehicle movement, three aspects are involved and stated as follows: (1) real-time detection of vehicle motion states; (2) dynamic analysis of dangerous driving behaviors; (3) correlation analysis and regularity discovery between vehicle motion state and dangerous driving behavior. The detection of vehicle motion state mainly involves the use of on-board monitoring equipment and the identification of motion state. The common equipment include vehicle on board diagnostics (OBD), camera, GPS, inertial sensor, smart phone, and so on. The selection and design of the detection method of vehicle motion state is related to the monitoring equipment and data type being used. In Huang (2011), the real-time recognition of vehicle Z-curve driving state based on image processing technology was proposed, which would automatically warn and provide feedback to the driver when the relevant image monitoring metric exceeded the preset threshold. Omerustaoglu et al. (2020) studied the driver’s distracted driving behavior by combining in-vehicle and image data using deep learning. Based on the theory of support vector machine (SVM), Jeong et al. (2013) recognized two kinds of driving behaviors, namely lane-changing and Z-curve driving using the data collected by the built-in 3-axis gyroscope of vehicle. DaeHan et al. (2019) proposed a system called ADDICT (Accurate Driver Detection exploiting Invariant Characteristics of smartphone sensors), which identifies the driver utilizing the inconsistency between gyroscope and magnetometer dynamics and the interplay between electromagnetic field emissions and engine startup vibrations. In order to evaluate the feasibility of ADDICT, four participants and three different vehicles by varying vehicle-riding scenarios are tested, and the evaluation results demonstrated that ADDICT identifies the driver’s smartphone with 89.1% average accuracy for all scenarios. Wu et al. (2013) used multiple sensors of vehicle monitoring cameras, 3-axis accelerometers and GPS receivers to collect vehicles’ motion parameters including lateral offset distance, relative distance, lateral/longitudinal acceleration, and speed. The recognition results for 7 common vehicle driving states (normal driving, acceleration, braking, left-turn, right-turn, curve driving, and vehicle following) verified that the hidden Markov model (HMM) had the best overall recognition rate.

The analysis of dangerous driving behavior mainly focuses on the classification of drivers’ driving styles. Some studies attempt to describe various types of aggressive driving behavior and develop their criteria (Tasca, 2000; Murphey et al., 2009; Abou-Zeid et al., 2011; Li et al., 2014; Carboni and Bogorny, 2015; Mãirean and Havãrneanu, 2018; Yang et al., 2019). In general, the classification algorithms of driving style can be divided into two categories: statistical method and machine learning method. Constantinescu et al. (2010) made use of vehicle-borne GPS data including GPS speed and acceleration to model and analyze driver’s driving style. In their research, the driving behaviors are divided into five types: non-aggressive, somewhat non-aggressive, neutral, moderately aggressive, and very aggressive. Hong et al. (2014) built a sensor platform composed of Android smartphones, OBD, and inertial measurement unit (IMU) for collecting driving behavior data including maximum, average and standard deviation, speed variation, longitudinal acceleration, lateral acceleration, speed, and throttle position of vehicles. Then the thresholds are determined that can equally divide these features of all samples into five discretized levels. Naive Bayesian classifier is utilized to model the relationship between driving characteristics and driving style. In Eboli et al. (2017), driving behaviors were divided into three types (safe, unsafe, and safe but potentially dangerous) by calculating the 50 and 80% speed and average speed.

For the classification of dangerous driving behavior, it is mainly realized by detecting driving events related to safety, such as acceleration, braking, and turning. In general, the classification of dangerous driving behavior can be divided into two categories: template-based matching method and threshold-based discrimination method. From the perspective of energy consumption, the acceleration–deceleration characteristics of three different driving behaviors are analyzed (Xing et al., 2020). Driving Habits Graph (DHG) (Chen et al., 2013), which indicates the significant changes of behavior according to a series of driving data, was proposed to simulate driving behavior and display the driving style intuitively. In their follow-up research (Chen et al., 2014), dangerous driving events were transformed into the attributed relational map (ARM), and then the two-way fuzzy attribute mapping and matching were used to compare the converted driving behavior with the template to determine whether it was a dangerous driving event.

In Johnson and Trivedi (2011), for all predefined driving events including right/left/U turn, aggressive right/left/U turn, and acceleration/deceleration/lane drastic change, the smartphone data were utilized to analyze these events and determine whether a driver’s behavior is normal or aggressive action through time series data matching and dynamic time warping (DTW). Based on vehicle-borne GPS and OBD data (Chen et al., 2019), a graphic modeling method was proposed for modeling individual driving behavior through the statistical method. Based on the assumption that drivers have specific driving habits, the typical driving modes are detected and extracted. Sorted by the frequency of these typical driving modes, a driving behavior diagram is finally constructed to directly explain the driver’s behavior characteristics. In Han and Yang (2009), the velocity, acceleration, and yaw angular velocity of vehicles are collected by an on-board black box for identification of four dangerous vehicles states including accelerating, decelerating, steep turn, and sudden lane change. Besides, a threshold division method based on different speed intervals is also proposed. After intensive study on the acceleration threshold of dangerous aggressive driving behaviors Johnson and Trivedi, 2011), concluded that the turning acceleration threshold for aggressive driving was 0.73 g, the emergency turning threshold was 0.74 g, the U-turn threshold was 0.91 g, the turning threshold of non-aggressive driving was 0.3 g, and the U-turn threshold was 0.56 g. In Bagdadi (2013), the threshold for determination of rapid acceleration and deceleration was ± 0.48 g ≈ 4.8 m/s^2^.

**FIGURE 1:**
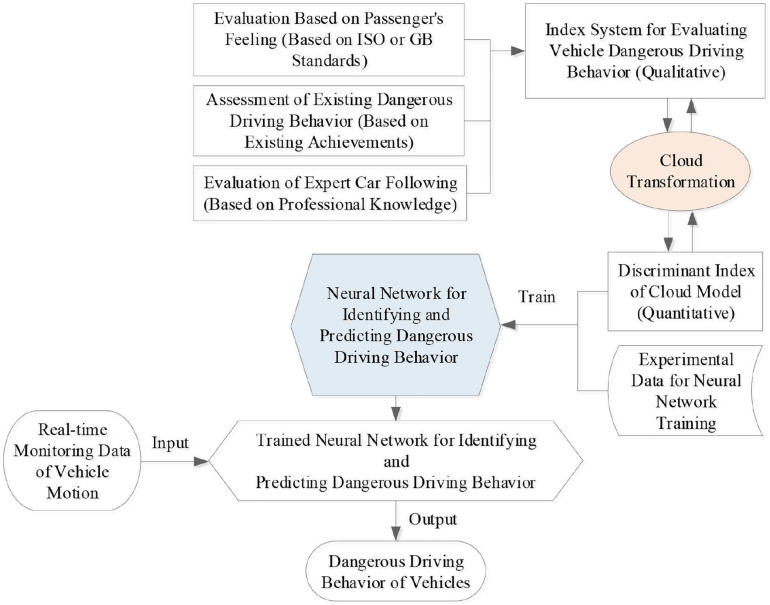
The overall framework of cloud model and Elman neural network (CM-ENN) model for dangerous driving behavior prediction.

From above literature review and analysis, we noticed that vehicle motion state data are almost collected by vehicle-mounted sensor units, such as GPS, accelerometer, etc. Current research on the dangerous vehicle state and driving behavior are most likely focused on the human driver and the operation of vehicles. Since the drivers are easy to be influenced by complex factors, it is difficult to find the personal characteristics of drivers. We think that the key to this problem lies in how to set up the evaluation index system of dangerous driving behavior scientifically, and to find an effective prediction algorithm that can convert these qualitative indicators into quantitative vehicle attitude data with high precision. In this context, this paper proposes a vehicle active safety monitoring and early warning method integrating driving behavior, passenger feeling, and vehicle status based on cloud model and Elman neural network (CM-ENN), which is illustrated in Figure 1. By following the indicators of vehicle ride comfort and passengers’ perception of vehicle motion in related ISO standards (ISO 2631-1:1997/AMD 1:2010, 2010) and the National Standards of China (National Technical Committee of Auto Standardization, 2004), a CM is built to set up correspondences between dangerous driving behavior and vehicle motion data. Because of the advantages of ENN in dealing with non-linear problems and dynamic information (Wang et al., 2021), a CM-ENN model is constructed where the CM is used to evaluate dangerous driving behavior incorporating passenger’s subjective feeling as well as vehicle motion data (Wang and Xu, 2012). The system was tested with the real data collected in vehicles running on some urban roads in Shenzhen City of China. Experimental results verified the effectiveness of the proposed method.

The remainder of this paper is organized as follows: section “Data Acquisition System” introduces the data acquisition and processing system of vehicle motion status. The calculation method of vehicle motion attitude is also discussed. In section “Cloud Model for Dangerous Driving Behavior Evaluation,” the CM theory is introduced and the details of setting up correspondences between dangerous driving behavior are explained. Section “CM-ENN, Prediction Method of Dangerous Driving Behavior” discusses the structure of ENN and the training process. Experimental results and analysis are presented in sections “Experimental Results and Analysis,” and “Conclusion” concludes the paper.

## Theory and Method

### Data Acquisition System

**FIGURE 2:**
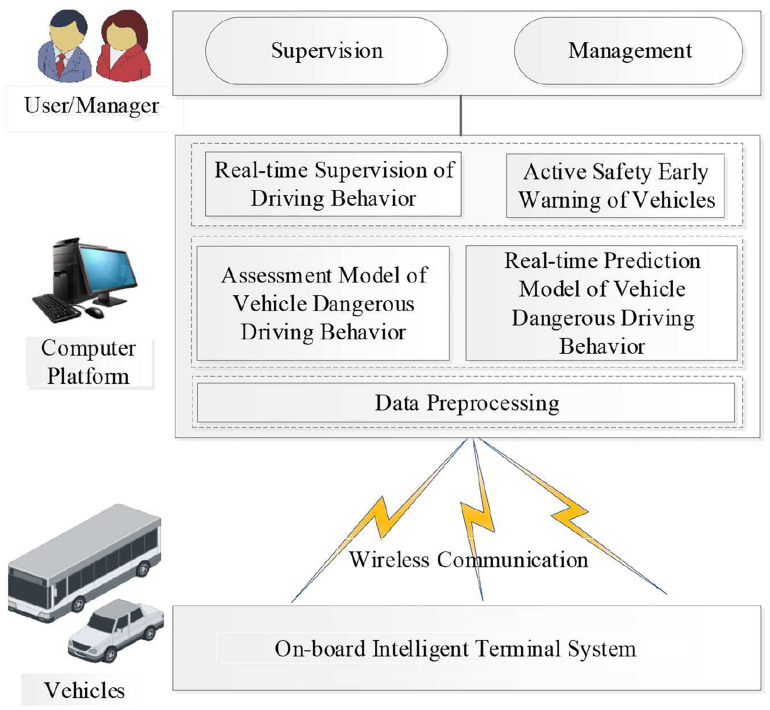
Real-time vehicle attitude monitoring system for dangerous driving behavior analysis.

In this paper, a real-time driving behavior monitoring and active safety early warning system is designed, as shown in Figure 2. The system consists of three parts: (1) Vehicle-borne intelligent terminal mainly includes vehicle-borne GPS, micro-electro-mechanical systems (MEMS) sensors, CAN-bus, and so on.

It is designed to realize the acquisition and transmission of real-time data of six degree of freedoms (DOFs) motion states and vehicle speed. (2) Computer platform: Main tasks for this part are as follows: First, to pre-process the collected data. Second, to provide real-time driving behavior information for users and managers through the CM discriminant criteria and the fast discriminant algorithm of vehicle driving behavior based on the CM-ENN model. The dangerous driving behavior is marked, warned, and stored. Third, the prediction model of vehicle motion attitude can be established based on the collected data so as to realize the active safety early warning of vehicle. (3) User/Manager: The main task is to evaluate the driver’s performance according to the processing results of the computer platform and to effectively curb the occurrence of dangerous driving.

**FIGURE 3:**
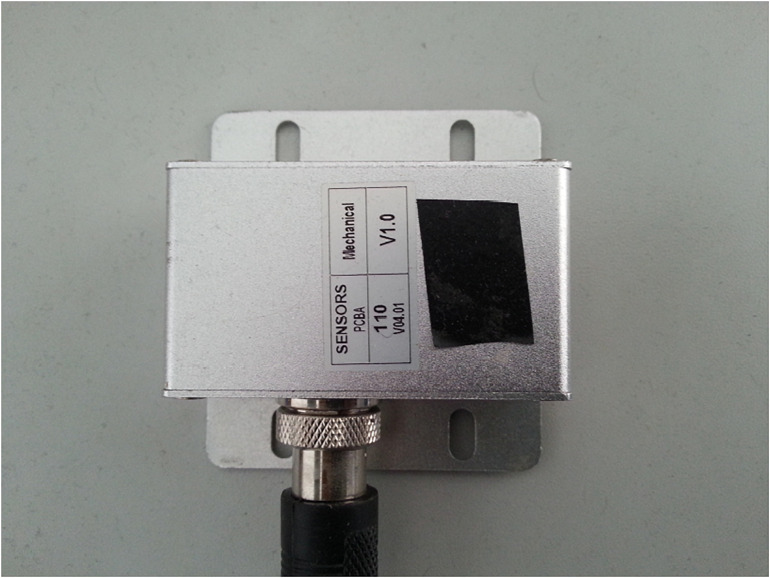
Inertial measurement unit (IMU) on vehicle.

**FIGURE 4:**
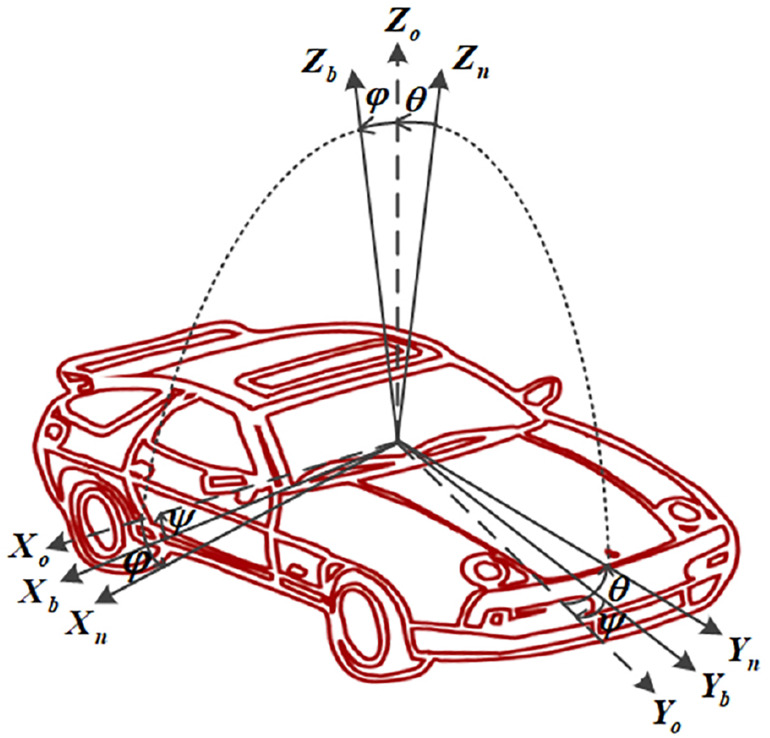
Transformation from body frame (OX_*b*_Y_*b*_Z_*b*_) to the navigation frame (OX_*n*_Y_*n*_Z_*n*_).

For the vehicle intelligent terminal, the six-axis MPU6500 (as shown in Figure 3) is selected as the integrated sensor of MEMS integrated with the accelerometer and gyroscope. The core processor of the main control module is STM32F207 VCT6, and the NEO-6M module is selected as the GPS module. The terminal is required to be installed at gravity center of the vehicle with three axes of the accelerometer aligned with the vehicle body. As shown in Figure 4, the forward direction of the vehicle corresponds to the positive direction of the *Y*-axis of the accelerometer, i.e., the longitudinal acceleration of the vehicle. The three axes angular velocity of the vehicle is measured by the gyroscope, and its direction is the rotation direction around the corresponding accelerometer axis. An on-board video driving recorder is also installed on the tested vehicle to record the whole process of video information during the testing process, which provides videos for the later data processing. The position, speed, and heading of the vehicle are acquired by output signal processing of accelerometer and coordinate transformation (Schmidt and Phillips, 2010). Generally, the motion state parameters of the carrier (such as attitude, speed, position, etc.) and the outputs of the sensor are not measured in the same coordinate system. Therefore, the coordinates need to be transformed by rotating around three coordinate axes. There are two coordinate systems shown in Figure 4. One is the carrier coordinate system (also known as system b, OX_*b*_Y_*b*_Z_*b*_) and the other is the navigation coordinate system (also known as system n, OX_*n*_Y_*n*_Z_*n*_). According to the rotation theorem in Euler navigation, the frame coordinates in carrier coordinate system can be transformed into the navigation frame coordinates by three consecutive rotations around different coordinate axes in a certain order. The transformation process can be expressed by:

(1) $$\left[\begin{array}{c} x_{n}\\ y_{n}\\ z_{n} \end{array}\right]=C_{b}^{n}\,\left[\begin{array}{c} x_{b}\\ y_{b}\\ z_{b} \end{array}\right]$$

where the transformation matrix $C_{b}^{n}$ is defined by the following equation:

(2) $$C_{b}^{n}=\left[\begin{array}{ccc} c\,o\,s\,\theta \,c\,o\,s\,\psi \; & c\,o\,s\,\theta \,s\,i\,n\,\psi \; & -s\,i\,n\,\theta \\ s\,i\,n\,\varphi \,s\,i\,n\,\theta \,c\,o\,s\,\psi \; & s\,i\,n\,\varphi \,s\,i\,n\,\theta \,s\,i\,n\,\psi \; & s\,i\,n\,\varphi \,c\,o\,s\,\theta \\ -c\,o\,s\,\varphi \,s\,i\,n\,\psi \; & -c\,o\,s\,\varphi \,s\,i\,n\,\psi \; & \\ c\,o\,s\,\varphi \,s\,i\,n\,\theta \,c\,o\,s\,\psi \; & c\,o\,s\,\varphi \,s\,i\,n\,\theta \,s\,i\,n\,\psi \; & c\,o\,s\,\varphi \,c\,o\,s\,\theta \\ +s\,i\,n\,\varphi \,s\,i\,n\,\psi \; & -s\,i\,n\,\varphi \,c\,o\,s\,\psi \; & \end{array}\right]$$

where yaw angle ψ, roll angle φ, and pitch angle θ are called Euler angles.

According to the fixed-point rotation theory of rigid body, there are three methods of solving attitude matrix including Euler angle method (known as three-parameter method), quaternion method (known as four-parameter method), and direction cosine method (known as nine-parameter method). Quaternion method is used in this paper to solve attitude matrix $C_{b}^{n}$ for the advantages of real-time performance and high precision. The fourth-order Runge-Kutta numerical integration method (Press et al., 2007) is applied to solve attitude parameters in quaternion and implement the transformation from system ***b*** to system ***n***.

### Cloud Model for Dangerous Driving Behavior Evaluation

In order to predict the dangerous driving behavior using the data collected from onboard sensors, it is crucial to create the evaluation criteria of the dangerous driving behaviors based on the vehicle motion states. On the one hand, the motion state of a vehicle at any time can be precisely measured quantitatively by various sensors in some metrics such as the speed, acceleration, and rotation angle; on the other hand, the dangerous driving behavior is actually a qualitative and conceptual description commonly used in traffic safety management, such as rapid acceleration, emergency braking, sharp turn, and so on. Therefore, mapping between vehicle motion state space and the dangerous driving behavior space is crucial. Based on the CM theory, this paper designs a CM for predicting dangerous driving behavior, which combines the vehicle driving states with the subjective feeling of passengers and establishes the mapping between the vehicle motion states and the dangerous driving behaviors.

Table 1 shows the root mean square (RMS) of the total acceleration of vehicle and the corresponding subjective feeling of human body, which to some extent reveals the relationship between the vehicle motion and human feeling. This classification standard can be used as a reference for evaluating dangerous driving behaviors. However, only comfort is considered in this table. Thus, we extend it with some other driving behavior description and use cloud transformation algorithm to build the numerical characteristics of dangerous driving behaviors that are provided as the targets for the ENN in the training process.

**TABLE 1:** The root mean square (RMS) of total acceleration and subjective feeling of the human’s body.

The RMS of the total acceleration a_ω_(m/s^2^)	Subjective feeling
0.315	Not uncomfortable
0.315 0.63	A little uncomfortable
0.5 1.0	Fairly uncomfortable
0.8 1.6	Uncomfortable
1.25 2.5	Very uncomfortable
>2.0	Extremely uncomfortable

#### Cloud Model Definition and Cloud Transformation Algorithm

CM is a cognitive model of bidirectional transformation between qualitative concept and quantitative data, which was proposed by Li et al. (2009). The basic concepts of CM are defined as follows: *Definition 1:* Let *U* be a quantitative domain expressed by exact numerical values, *C* be a qualitative concept on *U*, and *C* contains three numerical characteristics *(E_*x*_, E_*n*_, H_*e*_).* If a number *x* ∈ *U*, and *x* is a random realization of qualitative concept *C.* The certainty of *x* to *C* is *μ(x) (μ(x)* ∈ [0,1]), which is a random number with a stable tendency: *μ(x)* : [U → [0,1], *∀x* ∈ *U*, then the distribution of *x* on domain *U* is called CM. For a CM, each *x* is called a cloud droplet.

In Definition 1, three numerical characteristics of CM, *E_*x*_, E_*n*_*, and *H_*e*_*, are called expectation, entropy, and hyper-entropy, respectively, which represent a concept. Expectation *E*_*x*_ is the most representative concept or the typical sample in quantification of this concept; entropy *E*_*n*_ is the uncertainty measure of concept, which is determined by the randomness and fuzziness of the concept; hyper-entropy *H*_*e*_ is the uncertainty measure of entropy, which is determined by the randomness and fuzziness of the entropy. The number *x* depicts the randomness of quantitative values representing concepts, while *μ(x)* is the uncertainty of the number *x* belonging to a concept *C.*

The distribution differs for different CMs. Among them, the normal CM is the most important and of universal applications (Li et al., 2012). By forward cloud transformation (FCT) and backward cloud transformation (BCT), the CM realizes the mapping between qualitative concepts and their quantitative representations. The two algorithms of cloud transformation are displayed in Algorithms 1 and 2.

**Algorithm 1:** Forward Cloud Transformation (FCT)

**Input:** *Ex*,*En*,*He*,*n*

**Output:** *n* cloud droplets (*x*_*i*_,*μ*_*i*_),*i* = 1,2,…,*n*

Step 1: Generate a normal random number $E\,n_{i}^{\prime }=N\,O\,R\,M\,\left(E\,n,H\,e^{2}\right)$ with *En* as expected and *He*^2^ as variance 

Step 2: Generate a normal random number xi=N⁢O⁢R⁢M⁢(E⁢x,E⁢ni′2) with *Ex* as expected and E⁢ni′2 as variance 

Step 3: Calculate $\mu_{i}=e\,x\,p\,\left\{-\frac{{\left(x_{i}-E\,x\right)}^{2}}{2\,{\left(E\,n_{i}^{\prime }\right)}^{2}}\right\}$

Step 4: Calculate a cloud drop (*x*_*i*_,*μ*_*i*_) 

Step 5: Repeat steps 1–4 for *n* times to generate the required *n* cloud droplets drop(*x*_*i*_,*μ*_*i*_);

#### Index Based on Passenger’s Feeling

Based on lots of previous works and literatures on human body vibration, the International Standard Organization (ISO) has formulated ISO 2631 guidelines for the evaluation of human body’s response to whole body vibration. In ISO 2631-1:1997/AMD 1:2010 (2010) titled with “Evaluation of human exposure to whole-body vibration,” the exposure limit of human body is quantified in the main frequency range from 1 to 80 Hz during the transmission from solid surface to human body, and the human comfort feeling under different acceleration RMS is also demonstrated, as shown in Table 1. Generally in measurement of vehicle vibrations, the three-axis acceleration of IMU is used. Experiments show that the three-axle acceleration can effectively evaluate severities of vehicle vibration. The total acceleration is calculated by combining three-axis accelerations and used as the criteria for vibration evaluation, as described in the following:

**Algorithm 2:** Backward Cloud Transformation (BCT)

**Input:** *n* cloud droplets *x*_*i*_(*i* = 1,2,…,*n*)

**Output:** Expectation $\hat{E}\,x$, Entropy $\hat{E}\,n$ and Super Entropy $\hat{H}\,e$

Step 1: $\hat{E}\,x=\bar{X}=\frac{1}{n}\,\sum_{i=1}^{n}x_{i}$

Step 2: Random sampling grouping

**for** *i*←1 **to** *m* **do**

$|\begin{array}{l} \text{ for }j\;\leftarrow 1\text{ to }r\text{ do}\\ \text{ | Random sampling of n cloud droplet samples;}\\ \text{ end}\\ \;X_{i}=\{X_{i1},X_{i2},\text{…},X_{ir}\},{\bar{X}}_{i}=\frac{1}{r}\overset{r}{\underset{j=1}{\sum }}x_{ij} \end{array}$

**end**

$Y^{2}=\left\{Y_{1}^{2},Y_{2}^{2},\ldots ,Y_{m}^{2}\right\}$

$\hat{E}\,n^{2}=\frac{1}{2}\,\sqrt{4\,{\left(E\,Y^{2}\right)}^{2}-2\,D\,Y^{2}}$

Step 3: $\hat{H}\,e^{2}=E\,Y^{2}-\hat{E}\,n^{2}$

(1) For the vibration signal (three-axis acceleration), discrete Fourier transform (DFT) is applied to transforms it into the frequency domain using the following formula:

(3) $$X\,\left(f\right)=\sum_{n=0}^{N-1}x\,\left(n\right)\,e^{-j\,\frac{2\,\pi }{N}\,t}$$

where X(n) is a finite vibration signal with the length number *N* in the time domain that is the three-axis acceleration, and *X(f)* is the vibration signal in the frequency domain.

(2) Calculation of the RMS of one-third octave as well as the weight acceleration at the center of one-third octave. Formula of computing RMS of one-third octave is defined as:

(4) $$a_{i}=\sqrt{\frac{1}{f_{i\,u}-f_{i\,l}}\,\int_{f_{i\,l}}^{f_{i\,u}}X^{2}\,\left(f\right)\,df}\;i=1,2,3\,\ldots \,20$$

where **a*_*i*_* is RMS f one-third octave whose unit is *m/s^2^, f_*iu*_* is an upper cut-off frequency in the *i*^*th*^ frequency band, *f*_*il*_ is a lower cut-off frequency on the *i*^*th*^ frequency band, and *X(f)* is the acceleration signal in frequency domain.

Human body reacts differently to different frequency vibration in different directions, therefore, weighting factors are given in each frequency center to model the acceleration matching the real feeling of human body. ISO 2631-1:1997/AMD 1:2010 (2010) gives a frequency-weight table that indicate the center frequencies of one-third octave and the corresponding weighted factors for each axis. Thus, the weighed acceleration of each axis is simply calculated by looking up this table, as formulated by

(5) $$a_{w\,j}=\sqrt{\sum_{i=1}^{20}{\left(k_{i\,j}\,a_{i}\right)}^{2}}\;i=1,2,3\,\ldots \,20\;j=x,y,z$$

where a_*wj*_ is the weighed acceleration of each axis whose unit is m/s^2^, and k_*ij*_ is a weighted coefficient in the i^th^ one-third octave band for j axis.

According to the random input running test method of automobiles provided by the National Standards of China (National Technical Committee of Auto Standardization, 2004), acceleration of *X*-axis and *Y*-axis are weighted with 1.4, and *Z*-axis weighted with 1.0, and the total acceleration is calculated by

(6) aw=(1.4⁢aw⁢x)2+(1.4⁢aw⁢y)2+aw⁢z2

where *a*_*w*_ is the RMS of total acceleration, and *a_*wx*_, a_*wy*_, a_*wz*_* is the RMS of each axis computed by equation (5).

(3) The subjective feelings of comfort by human body are classified into six degrees, and the relationship between comfort and RMS of total acceleration *a*_*w*_ is shown in Table 1. Lots of research have proved that some dangerous driving behaviors such as sudden braking or sudden turning could also bring up uncomfortable feelings, which are classified into the category of “Very Uncomfortable” or “Extremely Uncomfortable.”

#### Comprehensive Cloud Model for Dangerous Driving Behavior Evaluation

Three measures including longitudinal acceleration *a_*y*_*, lateral acceleration *a_*x*_*, and total acceleration *a*_*w*_ of the vehicle are considered in evaluation of driving behaviors where *a*_*y*_ reflects the intensity of vehicle acceleration or deceleration, *a*_*x*_ indicates the intensity of the left turn or right turn of the vehicle, and the ISO recommend *a*_*w*_ as measure of passenger’s feeling of comfort in the riding process. In this paper, for simplicity we mainly adopt *a*_*y*_ which represents for the intensity of vehicle motion to corporate with *a*_*w*_ when evaluating the comprehensive state.

**FIGURE 5:**
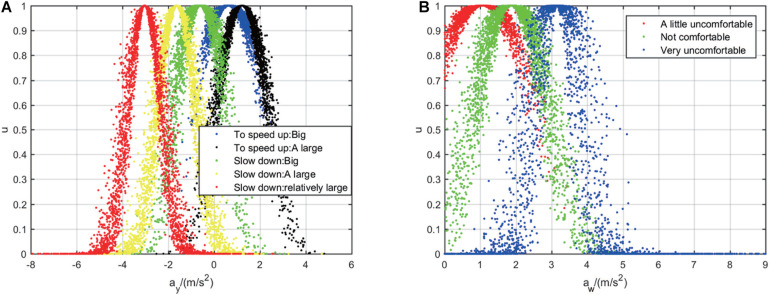
Acceleration of 1D cloud model: **(A)** Comparison of 1D cloud models with longitudinal acceleration; **(B)** comparison of 1D cloud models with total acceleration.

Based on CM theory and the input acceleration *a*_*y*_ and *a_*w*_*, this paper applied BCT to compute the numerical characteristics of the CMs for evaluating the intensity of vehicle motion and passenger’s feeling of comfort, as shown in Tables 2A,B. Then the FCT is applied to generate the corresponding one-dimensional CM maps, as illustrated in Figures 5A,B, respectively. There are five different color CMs in Figure 5 representing five degrees of intensity of vehicle motion, and three CMs in Figure 5B representing three degrees of comfort. The distributions of these one-dimensional CMs indicate the longitudinal acceleration *a*_*y*_ and the total acceleration *a*_*w*_ are very discriminative for vehicle motion states under different operating modes of drivers. In addition, there are overlapping part being observed between different droplet groups, which confirmed the CMs can also describe the uncertain part under certain states.

**TABLE 2:** Numerical characteristics of cloud models (CMs).

(A) CM of intensity				
	Numerical characteristics			Description
	Eχ_1_	Eη_1_	He_1_	Intensity
Speed up	0.6281 1.2202	0.5076 0.8511	0.1263 0.1637	Relatively large (black) Large (blue)
Slow down	−0.61 −1.635 −3.0326	0.7379 1.2357 1.4398	0.2832 0.274 0.3168	Relatively large(green) Large(yellow) Very large(red)

**(B) CM of comfort**				

**Numerical characteristics**				**Description**
**Eχ_2_**	**Eη_2_**	**He_2_**		**Comfort**

1.0682	0.5128	0.1472		A little uncomfortable (red)
1.8681	0.8931	0.2762		Not comfortable (green)
3.1463	1.5283	1.3423		Very uncomfortable (blue)

***(A)** In the column of “Intensity,” the color in the parentheses is correspond to the color of cloud in Figure 5A. **(B)** In the column of “comfort,” the color in the parentheses is correspond to the color of cloud in Figure 5B.*

According to concept division theory in CM, the dangerous driving behavior description is generated based on the input acceleration *a*_*y*_ and *a_*w*_.* These accelerations all consist of 3 states, and both comfort and intensity include 3 states. As a subset of all possible combinations, the driving behavior, therefore, is composed of 5 states and described by the comprehensive CM, as shown in Table 3. The main advantage of this definition is that it avoids direct judgment on the driving behavior based on the motion parameters retrieved by the motion sensor. The driving behaviors are essentially vague concepts and it is hard to determine the exact border of two behaviors. Incorporating human subjective feeling as well as building mapping from quantitative data space to concept space with CM make the judgment more flexible and as natural as what human does in real world. For example, if *a*_*y*_ is a positive value, which means the vehicle is speeding up. If the intensity is “relatively large” and the subjective feeling is “a little uncomfortable,” which means the action of speeding is not that bothering so the driving behavior is defined as “slow speeding.”

**TABLE 3:** Description of dangerous driving behavior with comprehensive cloud model.

	The input variable		The output variable		
	α_*y*_	α_*w*_	Intensity	Comfort	Driving behavior
Speed up	Big large	Big large	Relatively large Large	A little uncomfortable Not comfortable	Slow speeding Urgent to accelerate
Slow down	Big Relatively large Large	Big Relatively large Large	Relatively large Large Very large	A little uncomfortable Not comfortable Very uncomfortable	Slow speed reduction General slowdown Sharp slowdown

**FIGURE 6:**
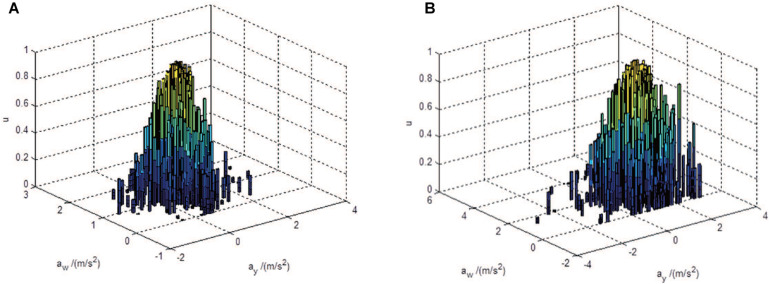
2D cloud model with different acceleration states: **(A)** Slow speeding; **(B)** urgent to acceleration.

For further description on different vehicle motion states, 1D CM can be extended to 2D by cloud transformation and concept escalation (Meng et al., 2010). Six numerical characteristics *(Exi,Eni,Hei,Ex2,Eri2,He2)* are used in this paper, where the expectation *Ex_1* and *Ex_2* are the best representation of the 2D concepts of vehicle status including vehicle motion intensity and comfort. The entropy En_1_ and En_2_ are the fuzzy measurements of vehicle status, which describes the coverage over 2D values. The hyper-entropy He_1_ and He_2_ depict the dispersion of cloud droplets, which are implicitly represented by the thickness of the 1D projection of the 2D CM. Taking vehicle accelerating as an example, the 2D CMs for two acceleration status, slow acceleration and rapid acceleration, are shown in Figures 6A,B.

As shown in Figures 5, 6, difference vehicle states have difference numerical characteristics of the corresponding CM. In order to make an intuitive comparison, by applying cloud computing the numerical characteristics of comprehensive CM are calculated based on the six numerical characteristics of 2D CM, as formulated by

(7) $$E\,x=E\,x_{1}+E\,x_{2}$$

(8) E⁢n=E⁢n12+E⁢n22

(9) H⁢e=H⁢e12+H⁢e22

In equation (7), Ex is comprehensive expectation, En is comprehensive entropy, and He is comprehensive hyper-entropy. The three numerical characteristics are the comprehensively representation of the qualitative concepts of different driving behaviors, as shown in Table 4.

**TABLE 4:** Numerical characteristics of comprehensive cloud model integrating the qualitative concepts.

Driving behavior	Ex	En	He
Slow speeding	1.6963	0.7215	0.1940
Urgent to accelerate	2.5372	0.9294	0.6600
Slow speed reduction	0.4582	0.8986	0.3192
General slowdown	0.2331	1.5247	0.3891
Sharp slowdown	0.1137	2.0997	1.3792

### CM-ENN, Prediction Method of Dangerous Driving Behavior

After quantifying the qualitative conceptual of dangerous driving behavior through evaluation of vehicle driving state and passengers’ subjective feelings by CM, a real-time identification model for dangerous driving behavior is designed, which is referred to as CM-ENN. The input of CM-ENN is the driving state data described before, usually in a sequence, and the target output is the predicted dangerous driving behavior. Inside the structure, ENN takes the charge of driving state prediction and CM takes the charge of determining which dangerous driving behavior it is. Considering the low-cost on-board platform with limited computing ability, for online training or prediction, the simple-structured ENN is an appropriate choice in this scenario.

#### The Structure and Algorithm Design of ENN

**FIGURE 7:**
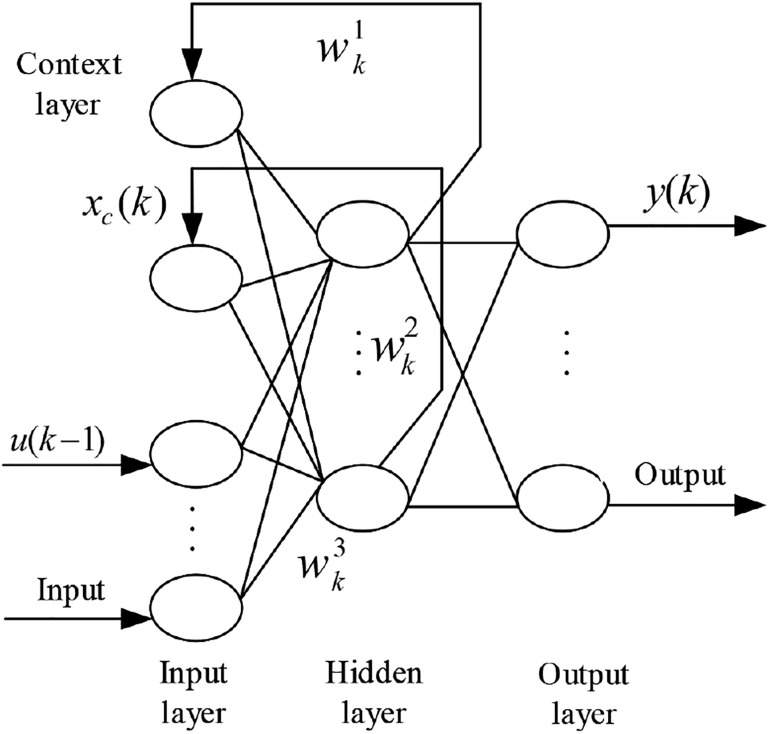
The structure of Elman neural network (ENN).

ENN was first proposed by Jeffrey L. Elman in 1990. Unlike static feedforward networks such as BP network and RBF network, Elman network is a dynamic local regression neural network. Different from classical BP network, this network has another feedback loop from the output of hidden layer to the input of this layer, which constitutes the “context layer” that retains information between observations. This type of network consists of an input layer, a hidden layer, an output layer, and a context layer. Typical structure of ENN is depicted in Figure 7. The input layer and output layer play the roles of signal transmission and linear weighting, respectively. The hidden layer is to take the previous output as its new input as well as the input of context layer. Thus, the context layer can be seen as a group of time-delay operators that enable the network with the capability of memorizing historical states. The transfer function of hidden layer can be linear or non-linear.

ENN’s non-linear space state can be expressed as follows:

(10) $$x\,\left(k\right)=f\,\left[w_{k}^{1}\,x_{c}\,\left(k\right)+w_{k}^{2}\,\mu \,\left(k-1\right)\right]$$

(11) $$x_{c}\,\left(k\right)=x\,\left(k-1\right)$$

(12) $$y\,\left(k\right)=g\,\left[w_{k}^{3}\,x\,\left(k\right)\right]$$

where *μ(k −* 1) is the external input, *x(k)* is the output of hidden layer, and *y(k)* is the output of the network. *w${}_{k}^{1}$, w${}_{k}^{2}$* and *w${}_{k}^{3}$* are the matrixes, which represent connection weights from the context layer to the hidden layer, the input layer to the hidden layer, and the hidden layer to the output layer, respectively. *f* and *g* are transfer functions of the hidden layer and the output layer.

In this paper, the Levenberg-Marquardt backpropagation learning algorithm is used in the training of ENN to adjust weights of each layer, and minimize the mean square error (MSE) between the network output and desired output, the energy function is expressed as

(13) $$E=\sum_{k=1}^{n}{\left[y\,\left(k\right)-d\,\left(k\right)\right]}^{2}$$

where *d(k)* is the desired output.

Assuming that the vehicle accelerations at the first *n* time points are taken to predict the acceleration at the next time point, the mapping function can be expressed as follows:

(14) $$x_{n}=f\,\left(x_{1},x_{2},\cdots \,x_{n-1}\right)$$

**FIGURE 8:**
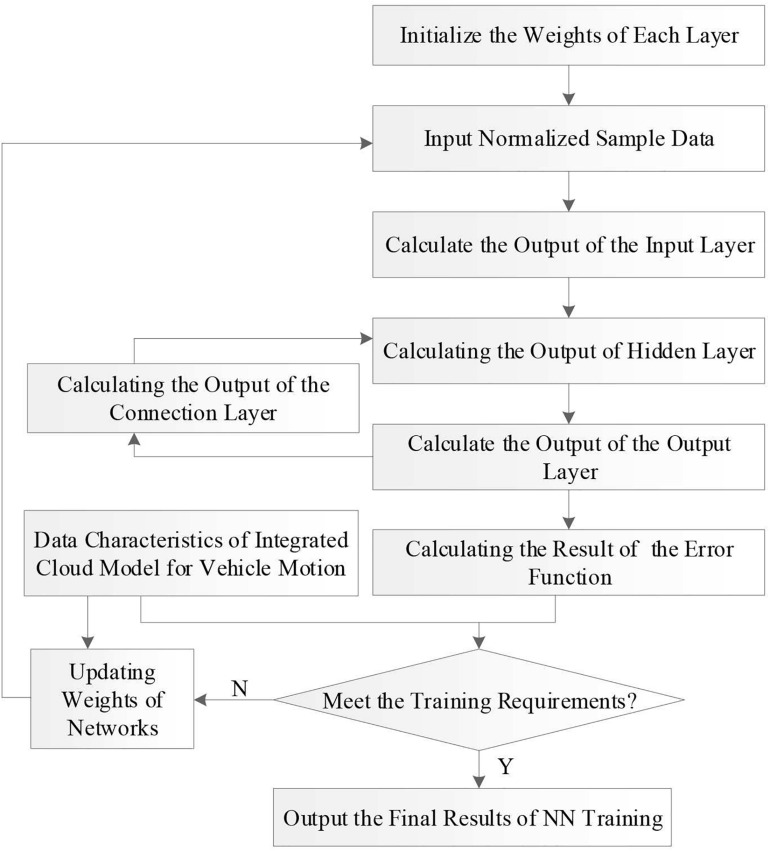
Flow chart of the cloud model and Elman neural network (CM-ENN) learning algorithm.

First, we construct the sample set. For the given vehicle acceleration data, the rule of cycle prediction is adopted to build the sample set, that is, taking the prediction of the previous step as the input of the next step, cycle down in turn. The driving behavior prediction therefore can be implemented by considering the historical and current data collected in vehicle motions instead of the specific information on driver’s driving style, road conditions, and so on. Second, the input data of neural network are normalized to [–1,1] using equation (15) and the network output is denormalized by equation (16).

(15) $$\hat{L}=\frac{2\,L-L_{m\,a\,x}-L_{m\,i\,n}}{L_{m\,a\,x}-L_{m\,i\,n}}$$

(16) $$L=\frac{1}{2}\,\left[\left(L_{m\,a\,x}-L_{m\,i\,n}\right)\,\hat{L}+L_{m\,a\,x}+L_{m\,i\,n}\right]$$

here **L_*min*_** and ***L*_*max*_** are the minimum and maximum of the data in the sample set. The input layer of ENN consists of 20 neurons, the output layer includes 1 neuron, and the neuron number in the hidden layer is set to 13. The flow chart of the CM-ENN learning algorithm is illustrated in Figure 8.

#### State Identification of Unlabeled Cloud Model

Whatever vehicle motion intensity feature or passenger feeling feature are used, they have been summarized into a CM described by a 3-element vector. And any data sequence can be applied to inverse CM generator to get the same length feature vector represented for an unlabeled CM. The identification of unlabeled CM can be seen as a similarity measurement problem of the CM. In this paper, we adopt the measurement called maximum boundary-based cloud model (MCM) (Yang et al., 2018), which generally is an overlapping area calculation method between two CMs based on integral. There are several types of CM similarity measurement, including integral-based and vector-based methods (cosine similarity). The main advantage of integral-based methods is that it can describe different roles of three individual feature values, instead of treating them the same in vector-based methods. Moreover, in MCM, the integral calculation, which is originally much more expensive than that of cosine-based methods, is transformed into standard normal distribution integral calculation, which can be pre-calculated. The simplification of computation is quite important for the real-time monitoring purpose. With MCM, unlabeled CM therefore can be compared to each type of baseline CM representing different states, as shown in Tables 2, 4, and the best-matched label is selected for it.

## Experimental Results and Analysis

### Data Acquisition

**FIGURE 9:**
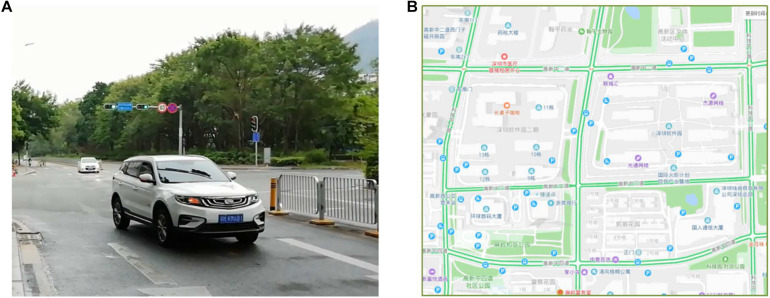
Testing data acquisition: **(A)** The vehicle for collecting testing data; **(B)** urban roads for collecting testing data.

**FIGURE 10:**
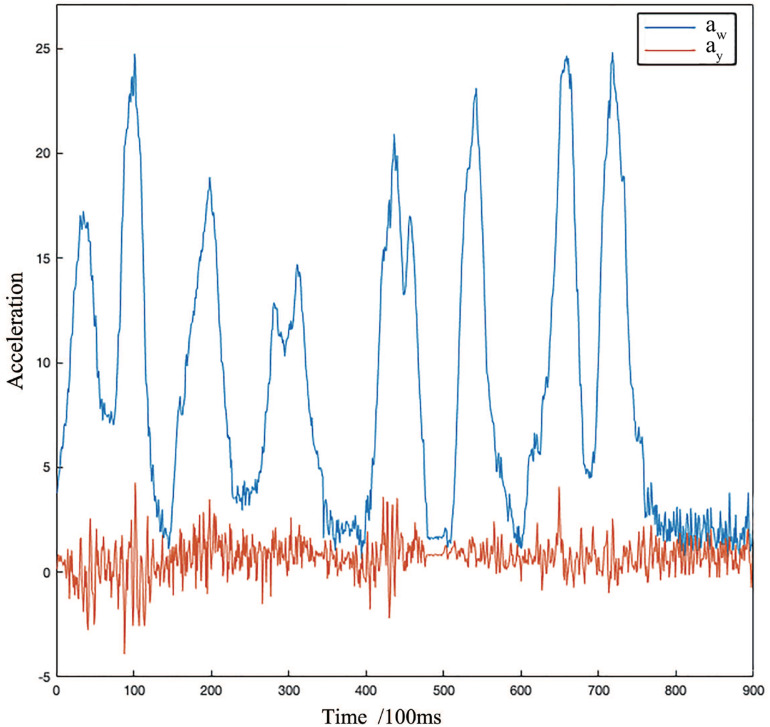
The response curve of RMS of total acceleration a_*w*_ and longitudinal acceleration a_*y*_.

In order to evaluate the performance of the proposed method, we used the on-board system described in previous section to collect the experimental data. The data acquisition area is located in the road network of Shenzhen Software Park Phase II on the north side of Nanshan Science Park, Shenzhen City, Guangdong Province, China (as shown in Figure 9). The data collection plan is carefully designed to ensure the randomness, autonomy, contingency, and suddenness of driving behavior. Besides, road safety is another concern in real-world data acquisition. In the data acquisition process, the ways of data recording include vehicle terminal recording, video recording, and manual observation recording. To ensure the objectivity and identicalness of manual observation, we invited three passengers to rate all driving behavior indicators, respectively. The true label is then determined by a simple on-site voting. The onboard IMU MPU6500 is utilized to collect velocity data and the frequency is 10 Hz. In this paper, 900 randomly selected historical data are taken as sample data. Using the coordinate transformation and evaluation method provided before, the change curves of total acceleration and longitudinal acceleration are obtained during the moving of the vehicle, as shown in Figure 10.

### Model Training and Experiments

Data sequences are treated as rolling inputs to train the models. In this experiment, one sample is defined as a 21-length sequence which is roughly 2 s in 10 Hz setting and the output is last value of this subsequence, which means the models are required to predict the value at next moment according to the previous 20-length sequence. Thus, a 900-length sequence can be separated into 880 samples. And in this experiment, 510 of them are used to train and the rest of them are used for validation.

To make a comparison, an ANN or called multi-layer neural network is designed which uses the same sample set, similar network architecture, learning algorithm, and target accuracy. The details of these two models are described in Table 5. The network structure is denoted by three numbers indicating the neuron number in input layer, hidden layer, and output layer.

**TABLE 5:** Comparison of results by Elman neural network and multi-layer neural network.

Model	Network structure	Learning algorithm	Number of training	Precision(%)
CM-ENN	20, 13, 1	Levenberg-Marquardt backpropagation	500	0.01
Artificial Neural Network (ANN)	20, 13, 1	Levenberg-Marquardt backpropagation	210	0.01

In order to compare and evaluate the prediction performance of different prediction methods, we adopt three measures including mean absolute error (MAE), mean square error (MSE), and root mean squared error (RMSE), which are defined as following equations:

(17) $$M\,A\,E=\frac{1}{n}\,\sum_{i=1}^{n}\left(\left|A_{t}-F_{t}\right|\right)$$

(18) $$M\,S\,E=\frac{1}{n}\,\sum_{t=1}^{n}{\left(A_{t}-F_{t}\right)}^{2}$$

(19) $$R\,M\,S\,E=\sqrt{\frac{1}{n}\,\sum_{t=1}^{n}{\left(A_{t}-F_{t}\right)}^{2}}$$

where A_*t*_ is the predicted value and F_*t*_ is the true value.

**FIGURE 11:**
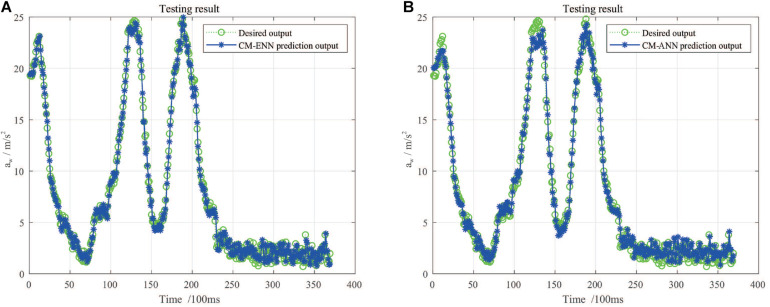
The predicted a_*w*_ values by two learning models and the recorded true values in a selected period of time: **(A)** Results of ENN; **(B)** results of ANN.

Figure 11 shows the predicted a_*w*_ values in a certain period of time by ENN and ANN. Table 6 shows the errors of training and testing by ENN and ANN. As shown in Figure 11, though the two models are capable to catch the time-series structure of input sequence, the ENN has lower validation error with all measures according to Table 6, which indicates that ENN performs better than ANN in this scenario and has better ability of generalization.

**TABLE 6:** The errors of training and testing.

		Comfort			Intensity			Driving behavior		
		MAE	MSE	RMSE	MAE	MSE	RMSE	MAE	MSE	RMSE
CM-ENN	Train	0.4184	0.0984	0.3137	0.5300	0.1673	0.4090	0.4653	0.1239	0.3520
CM-ANN		0.3718	0.0790	0.2811	0.5910	0.2209	0.4700	0.4074	0.1050	0.3240
CM-ENN	Validation	0.5692	0.1661	0.4076	0.5576	0.1789	0.4230	0.6526	0.2297	0.4793
CM-ANN		0.6072	0.1894	0.4352	0.5908	0.2014	0.4488	0.7705	0.3072	0.5543

After training, dangerous driving behavior can be predicted and judged by combining the prediction model and CM. The 880 samples obtained previously all have their ground truth label in comfort and intensity, which is described in Tables 2, 4. Leveraging the backward Algorithm 2, the predicted sequence of representation of comfort and intensity can be compacted into CMs and by using MCM, the cloud similarity measurement, these predicted CMs can be labeled and compared to their ground truth. The accuracy results are demonstrated in Table 7. Here, two types of predictions are made. One is using the models to predict next 1 s sequence, which has 10 values at the setting of 10 Hz, and another is 2 s, which has 20 values in total. The prediction accuracy of comfort, intensity, and comprehensive dangerous driving behavior are presented in the table. The results indicate that, as discussed before, though CM-ANN seems not bad when handling the comfort data, CM-ENN can much better catch the sequence structure. The errors accumulated by models will greatly affect the prediction accuracy of dangerous driving behavior, and CM-ENN has a more robust decay of accuracy as the length of predicted time increases. Besides, the prediction of dangerous driving behavior is not so accurate as that of comfort and intensity, probably because the comfort label is determined manually in our experiment, which may lead to incorrect correspondences with the true dangerous driving behavior pattern. Therefore, more accurate and interpretive comfort measures should be considered in future work.

**TABLE 7:** The accuracy of dangerous driving behavior prediction by Elman neural network (CM-ENN) and CM-ANN.

	Comfort		Intensity		Driving behavior	
Prediction Length(Second)	1 s	2 s	1 s	2 s	1 s	2 s
CM-ENN	0.8921	0.8746	0.9219	0.8873	0.8909	0.7979
CM-ANN	0.8370	0.7375	0.9357	0.8815	0.7910	0.7596

## Conclusion

Based on the analysis of existing research on dangerous driving behavior prediction, this paper puts forward a new CM-ENN model for predicting dangerous driving behavior by combining vehicle sensor data with passenger’s subjective feelings. The CM theory is introduced to implement transformation from quantitative space to qualitative space. Referring to the relevant standards, a comprehensive evaluation CM of dangerous driving behavior is constructed, which combines vehicle sensor data with passenger’s subjective feelings. To evaluate the performance of the proposed algorithm, the discriminant accuracy of this method and ANN are compared based on the same real world dataset and error control conditions. Experimental results verified the better prediction accuracy of the proposed CM-ENN model. This research provides a practical solution for safe driving in the development of automotive active safety management products. In addition, the driving behavior itself is also affected by many factors such as road, environment, weather, and so on. Many of these factors also have great uncertainty. In this paper, these factors are not considered enough and need to be studied in future work.

## Data Availability Statement

The datasets presented in this article are not readily available because the raw data supporting the research of this article will be made available to any qualified researcher by the authors. Requests to access the datasets should be directed to HX.

## Author Contributions

HX conceived the research project. HX, GL, YS, and JZ proposed the algorithm and performed discussion. JZ and YS developed the program and conducted the experiments and performed the English corrections. HX and GL wrote the manuscript. All authors reviewed and approved the submitted manuscript.

## Conflict of Interest

The authors declare that the research was conducted in the absence of any commercial or financial relationships that could be construed as a potential conflict of interest.
